# The Relationship between Occupation and Semen Quality 

**Published:** 2011-09-23

**Authors:** Mohammad Hossein Vaziri, Mohammad Ali Sadighi Gilani, Amir Kavousi, Marjan Firoozeh, Reza Khani Jazani, Ahmad Vosough Taqi Dizaj, Habibesadat Mohseni, Narges Bagery Lankarani, Mohammad Azizi, Reza Salman Yazdi

**Affiliations:** 1 Faculty of Health, Safety and Environment (HSE), Shahid Beheshti University of Medical Sciences, Tehran, Iran; 2 Department of Andrology, Reproductive Biomedicine Research Center, Royan Institute for Reproductive Biomedicine, ACECR, Tehran, Iran; 3 Department of Reproductive Imaging, Reproductive Biomedicine Research Center, Royan Institute for Reproductive Biomedicine, ACECR, Tehran, Iran; 4 Department of Epidemiology and Reproductive Health, Reproductive Biomedicine Research Center, Royan Institute for Reproductive Biomedicine, ACECR, Tehran, Iran

**Keywords:** Male infertility, Occupational Exposure, Semen Quality

## Abstract

**Background:**

Infertility can be a major concern for couples trying to conceive, and occupational hazards may constitute a main cause of infertility in men. Studies conducted throughout the world indicate that physical and chemical hazards in the workplace can have a negative impact on male fertility. The main objective of this study was to determine the frequency of occupational categories of men who attended an infertility clinic, and to evaluate the differences in the semen quality parameters among occupational categories.

**Materials and Methods:**

This cross-sectional study was conducted on 1164 males who were referred to the Infertility Research Center in Tehran for treatment of infertility in order to evaluate the effects of certain occupations on infertility. The participants were divided into several categories according to their occupations and evaluated by means of a questionnaire for duration of infertility, BMI, sperm count, percentage of normal sperm morphology and percentages of sperm with class A and class B motilities. Descriptive statistics, analysis of variance, and correlations were conducted using SPSS 16.0 for Windows.

**Results:**

There were no statistically significant differences in the mean sperm count or sperm morphology between occupational categories. Assessment of the differences in the frequency of sperm motility classes between occupational categories revealed a significant difference only in the frequency of sperm with class B motility. The lowest mean percentages of sperm with class B motility were seen in those involved in the transportation industry, a finding in agreement with a number of other researches.

**Conclusion:**

Our findings revealed an association between occupation and sperm motility. Since our study population was relatively small and in many cases exposures to work hazards were brief, a larger study group must be evaluated in order to support the preliminary results of this study.

## Introduction

Infertility can be a major concern for young couples, leading to a wide range of other problems including stress, anxiety, depression, family crisis and divorce. Infertile couples may spend large amounts of money and time on protracted infertility treatment, hoping to achieve the desired cure. In a study performed by Kamali et al. in 2007, it was found that out of 2492 infertile cases, registered from 1993 to 2001, 50.5% were related to male infertility and 11.6% were related to couple infertility (1). In a study performed by Yosefi at Mashhad University of Medical Sciences in 2001 on couples suffering from infertility, it was found that 35% of cases were caused by male factors and 13% by couple factors (2). In another study by Ghahramani and Ghaem in 2006, it was found that about half of all infertility cases are related to male factors (3). This study also emphasized that most infertile men do heavy, physically demanding jobs.

Based on statistics released by the WHO, the prevalence of infertility is 10-15%. This means that one out of six couples suffer from infertility, among whom 35-40% of cases are related to male infertility disorders and 20% related to couple factors. From these findings, it can be concluded that male fertility disorders play a leading role in half of all infertility cases. In a study performed by Vahidi et al., in 2005 (4), which included 28 provinces, it was revealed that 25% of Iranian couples experience primary infertility during their lifetime and 3.4% over a fixed period of time. Also, in a study by Molavi Nojomi et al. performed in west Tehran in 2000, the total prevalence of infertility was estimated at 12% (5).

Among the known etiologies leading to infertility are occupation and exposure to harmful environmental factors, both of which can be prevented. Preventing damaging occupational effects on the male reproductive system is a high priority for healthcare professionals and can be managed by promoting employee awareness and encouraging appropriate preventive measures when performing hazardous jobs. To this end, a list of hazardous jobs and factors has been provided and some of those jobs and several related ones were studied.

In some previously published studies it has been reported that exposure to chemical hazards such as lead and pesticides could result in abortion and a reduced birth rate (6-9). Regarding the effects of exposure to occupational chemical and physical hazards on semen quality parameters, results of some previous studies indicate a negative relationship between solvent or pesticide exposure and sperm motility and concentration (6, 10-12), and also a negative correlation between exposure to heat and sperm concentration, motility and normal morphology (13-15).

In a study by Sadighi et al. (16) in an Infertility Research Center in Iran, it is pointed out that some factors in the human environment, such as certain working conditions (occupational and environmental exposures), can put the human reproductive system at risk. This study showed that among 500 people, 164 (32.8%) were affected by known factors influencing spermatogenesis according to these identified subgroups: 36 persons (22%) affected by insecticides; 46 persons (28%) affected by solvents; 56 persons (34.1%) affected by heat; and 26 persons (15.9%) affected by a combination of these factors or others. In terms of occupation, 34 persons (6.8%) were farmers, 40 persons (7.8%) were drivers and 22 persons (4.4%) were welders. Occupational hazardous factors were high among farmers and their sperm counts were significantly lower. Painters were three times more affected by oligospermia; those exposed to heat and solvents followed in rank. Sperm mobility was significantly lower among welders (16).

## Materials and Methods

This cross-sectional study was conducted on men who attended an infertility clinic located in Tehran, Iran. The study consisted of questionnaires completed by trained interviewers to provide information about demographics, marital status, type and duration of infertility, occupational history including job title and task, and exposure to occupational physical hazards.

According to participants’ occupations and considering similar occupational exposures, twelve occupational categories were derived: Clerical, Sales, Agriculture, Painting, Services, Construction, Military, Mechanical, Transportation, Plastic Work, Metal Work and Electrical. Additionally, medical examinations by a urologist and semen analysis tests were performed for each participant. Semen specimens were assessed according to the WHO guidelines (1999) for volume, sperm concentration, progressive and non-progressive motility, and normal morphology (17). The semen analysis method in this study was computer-assisted semen analysis (CASA).

A total of 1164 patients were recruited by simple randomization from September 2009 to March 2010. Each subject signed an informed consent document after the goals of the study were fully explained. The Ethical Committee of Shahid Beheshti University of Medical Sciences approved the study. For statistical analysis SPSS 16 for Windows was used. Descriptive statistics were used to characterize the study population. The relationships between semen parameters, age and body mass index (BMI) were investigated using Spearman non-parametric correlation. Analysis of variance was used to compare semen parameters between occupational groups.

## Results

The means and standard errors of age, infertility duration and BMI of the participants were 33.83 ± 0.17 years, 6.23 ± 0.14 years and 26.01 ± 0.12, respectively. The frequencies of primary and secondary infertility were 89.7 and 10.3 percent, respectively. Sperm analysis test results revealed that the means and standard errors of the sperm count, percentages of normal sperm morphology and percentages of sperm with class A and class B motilities were 44.89 ± 0.96 million/ml, 7.36 ± 0.14, 8.6 ± 0.21 and 23.13 ± 0.34, respectively. Statistical analysis revealed a significant negative correlation between age and the mean percentage of sperm with class B motility (r =-0.13, p<0.001), but there were no statistically significant correlations between age and other sperm parameters, or between BMI and sperm parameters, because all participants’ BMIs were at the normal level.

Exposure to occupational physical hazards including heat, vibration, ionizing radiation and non-ionizing radiation were reported in 42.8% (n=498), 17.6% (n=205), 0.3% (n=4) and 39.1% (n=455) of the participants, respectively. Also, heavy physical exertion and prolonged sitting were reported in 43.7% (n=509) and 62.1% (n=723) of participants, respectively. The four most common occupational categories among participants were Clerical, Sales, Transportation and Construction with frequencies of 30, 13.8, 10.1 and 10 percent respectively, and the least common was Plastic Work with a frequency of 1.7 percent. There were no statistically significant differences in the mean sperm count or sperm morphology between occupational categories.

**Fig 1:**
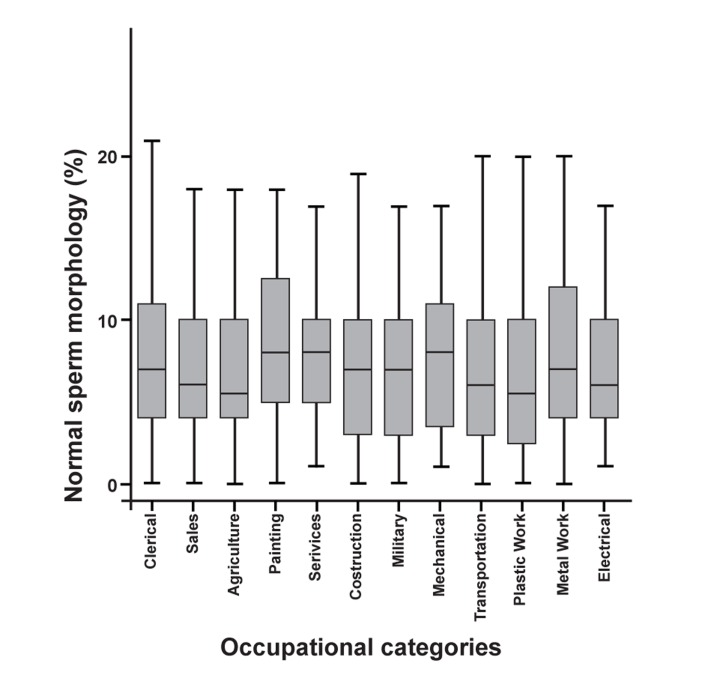
Normal sperm morphology of occupational categories (%)

**Fig 2:**
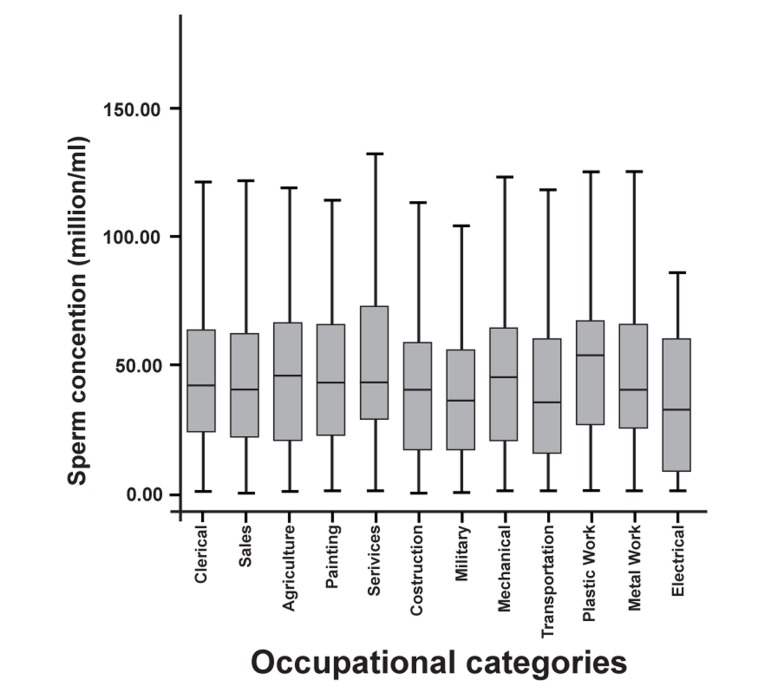
Sperm concentration of occupational categories (million/ml)

Percentages of normal sperm morphology and sperm concentration among occupational categories are shown in figures 1 and 2, respectively. Assessment of the differences in the frequency of sperm motility classes between occupational categories revealed a significant difference only in the frequency of sperm with class B motility (p=0.03).

**Fig 3:**
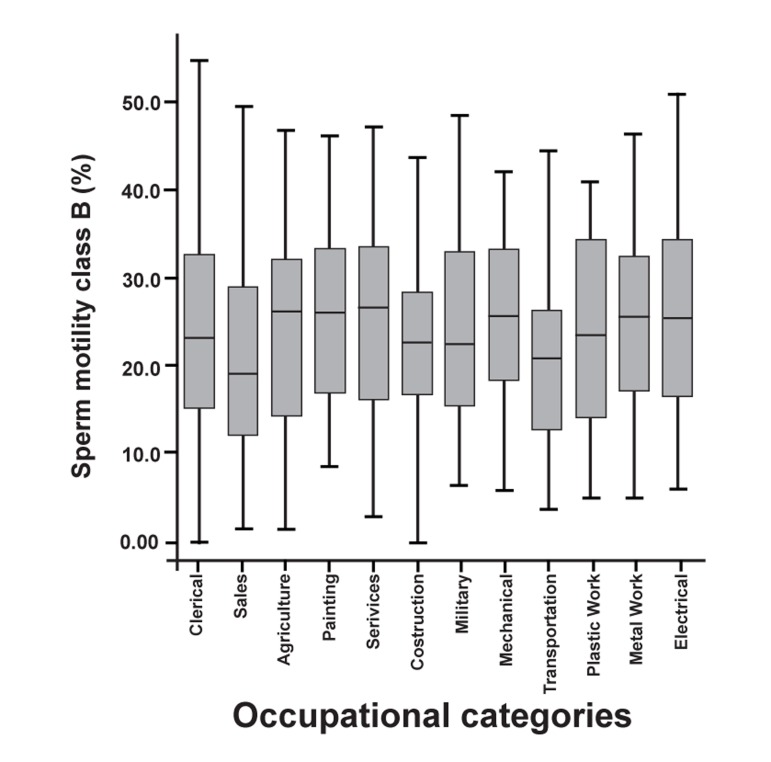
Sperm motility class A of occupational categories (%)

**Fig 4:**
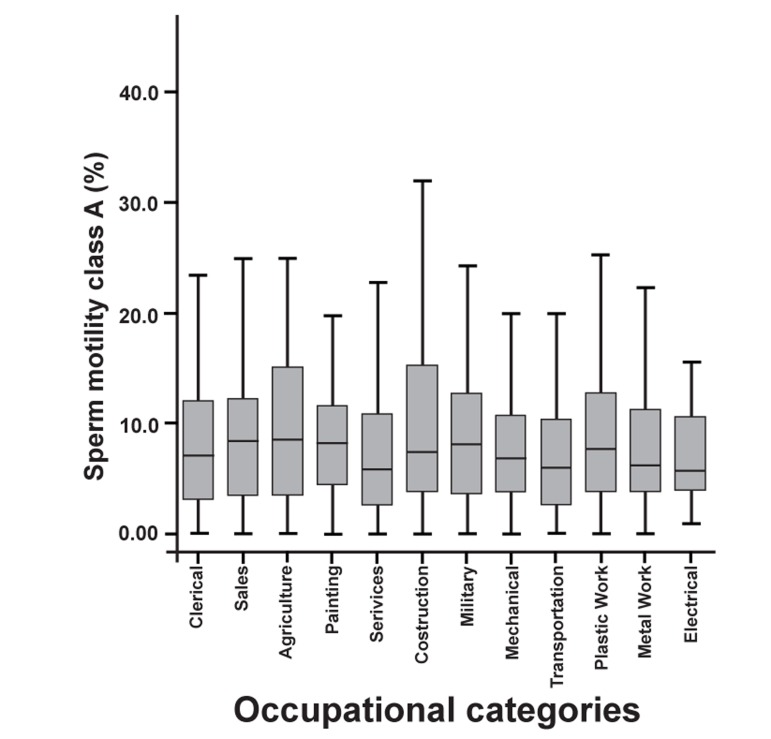
Sperm motility class B of occupational categories (%)

The highest and lowest mean percentages of sperm with class B motility were seen in the Electronics group (25.94 ± 2.5) and the Transportation group (20.26 ± 1.07), respectively. Figures 3 and 4 respectively show the percentages of sperm motility class A and B among the occupational categories. Details of semen quality parameters among the occupational categories and comparisons between them are shown in table 1.

**Table 1:** Comparison of semen quality parameters between the occupational categories


Semen quality parameters	Occupational categories												P-Value
	Clerical (n=349)	Sales (n=161)	Agriculture (n=55)	Painting (n=33)	Services (n=90)	Construction (n=116)	Military (n=73)	Mechanical (n=37)	Transportation (n=117)	Plastic work (n=20)	Metal work (n=85)	Electrical (n=28)	

**Volume of ejaculate (ml)Mean (SE)**	3.5 (0.09)	3.2 (0.13)	3.7 (0.29)	2.9 (0.26)	3.4 (0.18)	3.6 (0.16)	3.7 (0.19)	3.6 (0.3)	3.3 (0.17)	3.6 (0.43)	3.5 (0.17)	4.1 (0.33)	0.13
**Sperm Concentration (million/ml)Mean (SE)**	45.9 (1.78)	44.2 (2.32)	44.6 (4.3)	43.8 (4.7)	54.9 (4.43)	42.6 (3.17)	40.5 (3.71)	47.1 (5.84)	39.4 (2.72)	51.5 (7.25)	46.7 (3.4)	37 (5.27)	0.11
**Sperm Morphology (% Normal) Mean (SE)**	7.5 (0.25)	7.4 (0.354)	6.8 (0.596)	8.8 (0.825)	8.4 (0.522)	6.7 (0.444)	6.7 (0.502)	8.1 (0.981)	6.6 (0.492)	7.2 (1.315)	7.9 (0.549)	6.5 (0.753)	0.26
**Sperm Motility (% Class A)Mean (SE)**	8.6 (0.401)	9.1 (0.536)	10.1 (1.243)	8.5 (0.886)	7.6 (0.668)	9.9 (0.835)	8.9 (0.849)	8.5 (1.11)	7 (0.562)	8.9 (1.74)	8.4 (0.752)	7.4 (1.19)	0.23
**Sperm Motility (% Class B)Mean (SE)**	23.5 (0.61)	21.1 (0.94)	23.5 (1.69)	25.7 (1.95)	24.7 (1.274)	22.5 (0.94)	23.8 (1.43)	25.2 (1.73)	20.3 (1.07)	23.3 (2.85)	24.8 (1.13)	25.9 (2.56)	0.03


## Discussion

Workers can be exposed to a number of harmful physical, chemical and psychological factors in their working environment. During recent years, the various diseases and disorders caused by these stressors have drawn the attention of a number of researchers throughout the world. The effect of certain working hazards on the human reproductive system is one of the areas that have been studied and a number of reports concerning this have been published. In Iran however, a limited number of studies have been conducted on the growing problem of infertility. This study was undertaken with the goal of gaining a broader understanding of the factors in the working environment that can lead to decreased semen quality and related infertility in Iranian men. Understanding what constitutes a hazardous occupation in terms of its effect on fertility can be a major stepping stone to understanding how to deal with each factor and what types of preventive measures need to be taken.

In this study, information was obtained from 1164 men who reported problems with infertility. They were classified by occupation type including Clerical, Sales and Transportation, among others. The results indicate that the infertility rate among workers in the Transportation industry was highest in this study. The hazards that may cause this include being sedentary for long periods of time, vibration and exposure to heat. The semen analysis of this group showed that class B motility was lower than in other occupational groups (mean class B motility = 20.26 ± 1.07 and mean class A motility = 7.004 ± 0.56). The sperm morphology of this group was also lower compared to other occupations, but not significantly. Our results were similar to those reported by Figà-Talamanca et al. in a study in Italy on taxi drivers (18). Our results show that sperm counts among painters and construction workers compared to other occupations were lower which is also in agreement with results reported by Sadighi et al. (16).

In this research, 47.4% of participants reported that they had a stressful working environment.

42.8% were exposed to heat during the work period, similar to results reported by some previous studies (13, 16, 19). Rachootin and Olsen showed that exposing men to heat increased infertility (20). Hjollund et al. also showed that welders have low sperm counts (14).

Taking into account that 97.1% of our participants were from urban areas and only 2.9% from rural areas, we must assume that there are two possible factors leading to this difference. First, those men who live in rural areas have less access to the Infertility Research Center to seek treatment; second, men who live and work in rural areas are not exposed to industrial hazards on the same level as men in urban areas and have fewer problems with infertility; and third, the rate of rural and urban population are a determining factor in the percentages of infertility.

In relation to our work categories, 394 people (30%) of our participants were clerical workers and 161 (13.8%) in retail jobs. There is little documentation concerning the hazards associated with these occupations and we did not find any clinically significant correlation between the work environment and infertility in these groups.

Some previous studies have well documented the effects of chemical and toxic material on infertility and the motility and morphology of sperm (11, 16), and several studies show that smoking contributes to infertility. Our research, however, did not find a significant correlation between infertility and quality of sperm. We believe that the relatively small number of samples and the time of exposure could be the reasons for our findings and thus we could not thoroughly evaluate and analyze these effects. We recommend further research with samples taken from larger population groups who are directly exposed to chemicals for longer periods.

## Conclusion

In this study, we have determined the prevalence of men who were referred to the Infertility Research Center in Tehran suffering from infertility and the association of infertility to either their jobs or related factors, and then established the prevalence of risk factors. This study could help to recognize hazardous jobs and factors (both chemical and physical) in association with infertility in men and can be referred to as a resource for other analytical studies concerning all factors influencing infertility.

Considering the importance of occupational exposure and its hazardous effects on fertility which numerous studies have confirmed, it seems necessary to conduct a thorough descriptive study about the occupational factors influencing infertility among men who have been registered in the Infertility Research Center. To do this, we have also utilized the contributions of colleagues in other cities and the Ministry of Health to find the prevalence of infertility among men related to various occupations. A study could be designed to evaluate the prevalence of infertility in high-risk professions. Analytical studies can also be drawn based on such analysis and the relevant factors [such as Pb (lead), heat, solvents] and cases can be studied. Workers' safety and commitment to the safety principles in the workplace can keep infertility factors at a minimum level. Safety principles and standards have not been granted real status in Iranian workplaces and many harmful exposures, which are not considered problematic under the current standard, could be avoided with more thorough regulation.

Our findings support the results of previous studies regarding the association between occupation and sperm motility, particularly in the transportation category in which sedentary work is a common hazard. Further research is necessary to evaluate the observed associations in this study.
